# Risk factors associated with cardiovascular hospital admissions and all-cause mortality in cancer patients treated with immune checkpoint inhibitors

**DOI:** 10.1007/s00520-026-10983-6

**Published:** 2026-07-17

**Authors:** Joshua D. Bennetts, Jie Yu, Trent D. Williams, Andre Van der Westhuizen, Ina I. C. Nordman, Prajwol Shrestha, Rhonda Walker, Joerg Herrmann, Aaron L. Sverdlov, Doan T. M. Ngo

**Affiliations:** 1 School of Biomedical Sciences and Pharmacy, University of Newcastle, Callaghan, NSW 2308 Australia; 2 Hunter Medical Research Institute, Kookaburra Cct, New Lambton Heights Newcastle, NSW 2305 Australia; 3 Newcastle Centre of Excellence in Cardio-Oncology, The University of Newcastle, Hunter Medical Research Institute, Calvary Mater Newcastle, Hunter New England Health, Newcastle, NSW 2305 Australia; 4 Hunter New England Local Health District, Lookout Rd, New Lambton Heights, NSW 2305 Australia; 5 School of Nursing and Midwifery, University of Newcastle, Callaghan, NSW 2308 Australia; 6 Calvary Mater Hospital, Waratah, NSW 2298 Australia; 7 Department of Cardiovascular Diseases, Mayo Clinic, Rochester, MN 55902 USA; 8 School of Medicine and Public Health, University of Newcastle, Callaghan, NSW 2308 Australia

**Keywords:** Immune checkpoint inhibitors, Cardiovascular disease, Cardio-oncology, Risk factors, Hospital admission

## Abstract

**Purpose:**

Immune checkpoint inhibitors (ICIs) are highly effective cancer therapies. However, they are associated with considerable adverse cardiovascular (CV) outcomes. We investigated the risk factors for CV hospitalisations and all-cause mortality in oncology patients receiving ICIs.

**Methods:**

A retrospective cohort study of adult patients administered ICIs between 1st January 2010 and 1st January 2020 in New South Wales, Australia. Electronic medical records were accessed to obtain demographic and clinical data. Univariate analysis included Chi-squared test for categorical variables and Student’s *t*-test for continuous variables. Binary logistic regression was used for multivariate analysis. Mortality was analysed using Cox regression including (i) time-dependent Cox models with CV admission as a time-varying covariate and (ii) a 1-year landmark sensitivity analysis.

**Results:**

Out of 1,080 patients receiving ICIs during the study period, 340 patients (31.5%) had at least one CV hospital admission, and 763 patients (70.6%) had died by the end of the follow-up period. On multivariable analysis, prior history of heart failure and/or cardiomyopathy (*p* < 0.001), arrhythmia (*p* < 0.001) and ischaemic heart disease (*p* < 0.001) were independently associated with an increased risk of CV hospital admission by approximately fourfold, while CV hospitalisation during follow-up (*p* < 0.001) was independently associated with an increased risk of all-cause mortality. In a time-dependent Cox model (*n* = 928; excluding CV admissions with missing admission timing), CV admission was associated with higher subsequent mortality (HR 3.29 [95% CI 2.70–4.02]; *p* < 0.001). In a 1-year landmark sensitivity analysis among patients alive at 365 days (*n* = 533), CV admission within 1 year was associated with higher subsequent mortality (HR 2.36 [95% CI 1.61–3.45]; *p* < 0.001).

**Conclusion:**

In cancer patients treated with ICIs, pre-existing CV risk factors significantly increase the risk of CV hospitalisations and all-cause mortality. Appropriate CV risk monitoring and management should be considered for people with cancer receiving ICI therapy.

**Supplementary Information:**

The online version contains supplementary material available at 10.1007/s00520-026-10983-6.

## Introduction

Immune checkpoint inhibitors (ICIs) have emerged as highly effective targeted anticancer therapies. The cornerstone ICI classes—cytotoxic T-lymphocyte-associated antigen 4 (CTLA-4) inhibitors and programmed death 1 (PD-1)/programmed cell death-ligand 1 (PD-L1) inhibitors—are highly effective at suppression of cancer cells’ ability to avoid detection of a host’s immune system. [1, 2]. ICIs are ubiquitously used for the treatment of melanoma and non-small cell lung cancer (NSCLC), with increased utilisation for numerous other cancers [3], including breast cancer, Hodgkin’s lymphoma, colorectal cancer, and renal cell carcinoma [4, 5]. However, as expanding use of ICIs continues to revolutionise cancer treatment, cardiovascular (CV) events secondary to ICI exposure and subsequent development of cardiovascular disease (CVD) become a significant clinical issue. This is especially concerning in cancer patients with pre-existing history of CVD as they appear to be at a higher risk of cardiotoxicity [6].

The identification of CV events related to ICIs has increased over the years following the first case-specific report of pembrolizumab-induced myocarditis in 2015 [7]. Fulminant ICI-related myocarditis has since been widely documented [8] and is associated with high mortality, especially in those treated with combination therapy compared to monotherapy [9, 10]. While myocarditis has garnered the most attention, it is important to note that other CV adverse effects of ICIs are more common, including accelerated coronary atherosclerosis/acute coronary events and cardiomyopathy and heart failure (HF) in the absence of myocarditis [11–13]. These can occur any time during treatment but can also occur after completion of ICI therapy [14].

While there has been increasing literature regarding risks and prognostic factors for ICI-related myocarditis [15] there is scant literature available pertaining to the underlying risk factors associated with other forms of CV morbidity and mortality for those receiving ICI therapy. We therefore aimed to characterise baseline cardiovascular risk factors associated with cardiovascular hospitalisation and all-cause mortality in a large Australian real-world cohort treated with immune checkpoint inhibitors, with a focus on clinically encountered hospitalisation-level events beyond myocarditis.

## Methods

### Study population and data collection

This was a retrospective cohort study of adult oncology patients treated with ICIs between 1st January 2010 and 1st January 2020 across the Hunter New England Local Health District (HNELHD), New South Wales, Australia. The HNELHD services a large region covering 131,785 square kilometres, with population approximately 1,000,000 [16]. Ipilimumab was the first ICI registered by the Australian Therapeutic Goods Administration in July 2011 for unresectable stage III or stage IV malignant melanoma [17] and later listed on the Pharmaceutical Benefits Scheme in August 2013 [18]. This was considered when establishing the data extraction timeline.

Patients were identified and demographic and oncological data extracted using ARIA Oncology Information System. Hospital electronic medical records were accessed through the HNELHD Clinical Applications Portal (CAP) for patient follow-up until 31st December 2022 or their time of death. Rigorous data extraction methodology was employed to pre-define patient characteristics and CV outcomes to standardise data collection and minimise missing data. Patient electronic medical records were meticulously reviewed to extract relevant information pertaining to our outcomes. Collected information included demographic information, medication use and comorbidities pre- and post-ICI treatment, immune-related adverse effect (irAE) presentations, mortality and date of death where applicable.

CV hospital admission data, including date of admission, was obtained from Institutional Cardiac and Stroke Outcomes Unit database [19] and electronic medical record extraction. Date of ICI commencement was considered baseline for CV hospital admission, with patients considered event-free until their first CV admission. Hospital admissions data was extracted until patient death or conclusion of the study follow-up period. Additional information was obtained from electronic medical records via CAP. CV admission was defined according to coding based on the International Statistical Classification of Diseases and Related Health Problems, 10th Revision (ICD-10), which included hypertensive heart and renal disease, ischaemic heart disease (IHD), valvular heart disease, myocarditis, pericarditis, cerebrovascular disease (CVaD) excluding transient cerebral ischaemic attacks, pulmonary hypertension, HF and/or cardiomyopathy, arrhythmias and other conduction disorders. For admissions, cardiovascular diagnoses were defined using the principal discharge ICD-10 code where available. CV risk factors included overweight/obese status (body mass index (BMI) ≥ 25 kg/m^2^), male sex, age > 65 years, smoking history, hypertension, diabetes mellitus (DM), and dyslipidaemia. Pre-existing CVD included established HF and/or cardiomyopathy, arrhythmia, including atrial fibrillation, atrial flutter, and ventricular tachyarrhythmias, and any degree IHD, CVaD, peripheral vascular disease (PVD), or venous thromboembolism (VTE) by the time of ICI treatment.

### Statistical analysis

Categorical variables are reported as numbers and percentages, and continuous variables are reported as mean ± standard deviations or median (interquartile range (IQR)). Comparisons were performed between patients who did versus those who did not have a CV admission after commencement of ICI therapy, as well as those deceased or alive at the end of follow up. Baseline patient clinical characteristics (demographic data, medication use, and pre-existing comorbidities) were compared using Student’s *t*-test for continuous variables, and Chi-squared test or Fisher’s Exact Test for categorical variables. Backwards stepwise binary logistic regression models for CV admissions and all-cause mortality—iteratively removing the least significant variable at each step until the model’s predictive power ceases to significantly decrease—were then constructed to generate odds ratios (OR) with 95% confidence intervals (CIs). Variables for each model were selected based on a combination of statistical significance and/or clinical relevance. Multicollinearity was assessed for variables included in multivariable models to minimise the risk of unstable coefficients and inflated variance, leading to result interpretation issues. Tests for multicollinearity included variance inflation factor and tolerance, with thresholds of > 10 and < 0.01 established respectively, with variables removed from multivariable models if thresholds were met. Unadjusted and adjusted hazard ratios (HRs), stratified according to baseline clinical characteristics, were calculated from Cox proportional hazard models. Kaplan–Meier curves were generated for survival analysis. Analyses were performed using IBM SPSS Statistics v28 (IBM, Armonk, NY, USA), and *p* < 0.05 was considered statistically significant. Statistical tests were performed based on complete-case analysis.

To address survival-time (immortal-time) bias arising from CV hospitalisation as an intermediate event, we fitted Cox models with CV admission as a time-varying covariate (0 before first CV admission, 1 thereafter) using a start–stop (counting process) data structure. Patients without a CV admission contributed a single interval; patients with a dated first CV admission contributed two intervals (pre- and post-admission). Time-dependent analyses included all patients without CV admission and those with a dated first CV admission (*n* = 928); patients with CV admission but missing admission timing were excluded from time-dependent models. Simon–Makuch plots were generated to visualise survival according to the time-varying occurrence of first CV admission. We also performed a 1-year landmark sensitivity analysis, restricting to patients alive at 365 days after ICI initiation, and modelling subsequent mortality from day 365 onwards according to whether a CV admission occurred within the first year. To address competing risk considerations for time to first CV admission, we fitted a cause-specific Cox proportional hazards model for time to first CV admission (censoring at death or end of follow-up) within the same analytic set requiring dated first CV admission timing.

## Results

### Population demographics

A total of 1,129 patients were coded to have received ICIs between 1st January 2010 and 1st January 2020 throughout the HNELHD. An additional 44 patients were subsequently excluded from the sample population as they were prescribed daratumumab monotherapy — a CD38 monoclonal antibody. Lastly, 5 patients were also excluded from the final analysis due to incomplete treatment data, yielding a total of 1,080 patients that were included in the final analysis. Consort diagram is presented in Supplementary Fig. 1.

Table 1 displays patient characteristics for the whole patient cohort. The mean age of patients at their first dose of ICI was 66.9 (± 11.7) years, with median follow-up time of 1.4 years following initial ICI exposure. Additional follow-up data are presented in Supplementary Tables 1, 2, and 3. Almost two thirds (63.4%) of patients were male, with BMI of 25.8 kg/m^2^ (IQR of 22.35–29.82). The most prevalent primary cancer types were melanoma (38.7%; including melanoma in situ) and thoracic malignancies (30.3%; including lung cancer and mesothelioma). Regarding cancer staging, over two-thirds of our cohort (68.9%) had stage IV disease. Nivolumab was the most widely used first-line ICI treatment (44%) followed by pembrolizumab monotherapy (39.6%) and ipilimumab in combination with nivolumab (7.8%; includes those enrolled in placebo-controlled clinical trials). The commonest CV comorbidities were hypertension (52.9%), dyslipidaemia (38.2%), and prior IHD (17.8%). More than half of patients (58.3%) had three to five CV risk factors, and 139 patients (12.9%) had six or more CV risk factors.

**Table 1:** Baseline demographics of patients treated with immune checkpoint inhibitors for their cancer

Characteristic	General cohort *n*= 1,080 (%)
**Patient demographics**	
Age (years), first immune checkpoint inhibitor dose, mean (± SD)	66.9 (± 11.7)
Body mass index (kg/m^2^), median (IQR)	25.8 (22.4–29.8)
Gender (male)	685 (63.4)
Smoking status (active or ex-smoker)	669 (61.9)
**Comorbidities**	
Anxiety/depression	233 (21.6)
Arrhythmia	140 (13.0)
Asthma	93 (8.6)
Chronic kidney disease	65 (6.0)
Chronic obstructive pulmonary disease	230 (21.3)
Diabetes mellitus (type I or type II)	226 (20.9)
Dyslipidaemia	413 (38.2)
Heart failure and/or cardiomyopathy	55 (5.1)
Hypertension	571 (52.9)
Previous peripheral vascular disease	41 (3.8)
Previous venous thromboembolism	113 (10.5)
Prior cerebrovascular disease	92 (8.5)
Prior ischaemic heart disease	192 (17.8)
**Cancer type**	
Lung cancer and mesothelioma	327 (30.3)
Melanoma (including in situ)	418 (38.7)
Other cancer types	269 (24.9)
Unknown/not reported	66 (6.1)
**Cancer staging**	
Stage I	15 (1.4)
Stage II	9 (0.8)
Stage III	90 (8.3)
Stage IV	744 (68.9)
Unknown/not reported	222 (20.6)
**First-line ICI treatment**	
Ipilimumab + nivolumab (including placebo-controlled clinical trials)	84 (7.8)
Nivolumab	475 (44.0)
Pembrolizumab	428 (39.6)
**Other systemic anticancer treatments**	
Carboplatin	350 (32.4)
Dabrafenib + trametinib	76 (7.0)
Gemcitabine	267 (24.7)
Paclitaxel	77 (7.1)
**Medications**	
ACEi/ARB	489 (45.3)
Antiarrhythmic	55 (5.1)
Antidepressant	228 (21.1)
Antiplatelet	273 (25.3)
Antipsychotic	32 (3.0)
Beta-blocker	248 (23.0)
Calcium channel blocker	229 (21.2)
Direct-acting oral anticoagulant	96 (8.9)
Insulin	66 (6.1)
Loop diuretic	78 (7.2)
Metformin	161 (14.9)
Mineralocorticoid receptor antagonist	33 (3.1)
Sodium-glucose co-transporter 2 inhibitor	19 (1.8)
Statin	424 (39.3)
**Number of cardiovascular risk factors**	
Less than three	300 (27.8)
Three to five	630 (58.3)
Six or more	139 (12.9)

Number of cardiovascular risk factors is based on male sex, age over 65 years, body mass index 25 kg/m^2^ or over, diagnosis of hypertension, dyslipidaemia, diabetes mellitus, and smoking status of patients receiving immune checkpoint inhibitors examining predictors of cardiovascular admissions

*SD* standard deviation, *IQR* interquartile range, *ICI* immune checkpoint inhibitor, *ACEi/ARB* angiotensin converting enzyme inhibitor/angiotensin receptor blocker

### Cardiovascular admission analysis

A total of 340 patients (31.5%) had CVD included as a principal diagnosis during at least one hospital admission following their first dose of ICI. Patients’ demographics stratified according to CV-related hospital admissions are compared on a univariate analysis as displayed in Table 2. Reasons for these CVD hospital admissions are displayed in Supplementary Table 4, with IHD (19.4%), VTE (19.1%) and atrial fibrillation or atrial flutter (15.6%) being the most common. Patients hospitalised for a CV condition, following commencement of ICI, were typically older (mean age 70.2 vs 65.4 years, respectively; *p* < 0.001), more likely to be male (69.7% vs 60.5%; *p* = 0.004) and overweight/obese (27.3 kg/m^2^ vs 26.3 kg/m^2^, respectively; *p* = 0.007), compared to those who did not have a CV hospitalisation. Median days to first CVD-related hospitalisation was 190.5 days (IQR 61.25–653.75); though there was considerable missing data pertaining to date of CVD hospital admissions. Of the 340 patients with at least one CV admission, 188 (55.3%) had a usable time to first CV admission and 152 (44.7%) had missing admission timing. Accordingly, time-to-event analyses requiring admission timing (time-dependent Cox and cause-specific Cox) included 928 patients (740 with no CV admission and 188 with dated first CV admission).

**Table 2:** Univariate analysis of patient demographics stratified according to cardiovascular-related hospital admissions

Characteristic	CV admission *n *= 340 (%)	No CV admission *n *= 740 (%)	*p*-value
**Patient demographics**			
Age (years), first ICI dose, mean (± SD)	70.2 (± 9.8)	65.4 (± 12.1)	** < 0.001**
Body mass index (kg/m^2^), median (IQR)	27.3 (22.9–30.5)	26.3 (22.0–29.5)	**0.007**
Gender (male)	237 (69.7)	448 (60.5)	**0.004**
Smoking status (active or ex-smoker)	243 (71.5)	426 (57.6)	** < 0.001**
**Comorbidities**			
Anxiety/depression	80 (23.5)	153 (20.7)	0.301
Arrhythmia	83 (24.4)	57 (7.7)	** < 0.001**
Asthma	27 (7.9)	66 (8.9)	0.642
Chronic kidney disease	30 (8.8)	35 (4.7)	**0.013**
Chronic obstructive pulmonary disease	100 (29.4)	130 (17.6)	** < 0.001**
Diabetes mellitus (type I or type II)	100 (29.4)	126 (17.0)	** < 0.001**
Dyslipidaemia	165 (48.5)	248 (33.5)	** < 0.001**
Heart failure and/or cardiomyopathy	41 (12.1)	14 (1.9)	** < 0.001**
Hypertension	202 (59.4)	369 (49.9)	**0.004**
Previous peripheral vascular disease	24 (7.1)	17 (2.3)	** < 0.001**
Previous venous thromboembolism	40 (11.8)	73 (9.9)	0.338
Prior cerebrovascular disease	38 (11.2)	54 (7.3)	**0.045**
Prior ischaemic heart disease	127 (37.4)	65 (8.8)	** < 0.001**
**Cancer type**			
Lung cancer and mesothelioma	102 (30.0)	225 (30.4)	0.943
Melanoma (includes in situ)	133 (39.1)	285 (38.5)	0.893
Other cancer types	83 (24.4)	186 (25.1)	0.821
Unknown/not reported	22 (6.5)	44 (5.9)	0.785
**Cancer staging**			
Stage I	6 (1.8)	9 (1.2)	0.576
Stage II	3 (0.9)	6 (0.8)	1.000
Stage III	24 (7.1)	66 (8.9)	0.344
Stage IV	243 (71.5)	501 (67.7)	0.229
Unknown/not defined	4 (1.2)	23 (3.1)	0.061
**First-line ICI treatment**			
Ipilimumab + nivolumab (includes placebo-controlled clinical trials)	19 (5.6)	65 (8.8)	0.086
Nivolumab	139 (40.9)	336 (45.4)	0.167
Pembrolizumab	150 (44.1)	278 (37.6)	**0.045**
**Other systemic anticancer treatments**			
Carboplatin	117 (34.4)	233 (31.5)	0.363
Dabrafenib + trametinib	16 (4.7)	60 (8.1)	0.054
Gemcitabine	89 (26.2)	178 (24.1)	0.449
Paclitaxel	20 (5.9)	57 (7.7)	0.310
**Medications**			
ACEi/ARB	185 (54.4)	304 (41.1)	** < 0.001**
Antiarrhythmic	35 (10.3)	20 (2.7)	** < 0.001**
Antidepressant	78 (22.9)	150 (20.3)	0.336
Antiplatelet	136 (40.0)	137 (18.5)	** < 0.001**
Antipsychotic	8 (2.4)	24 (3.2)	0.562
Beta-blocker	135 (39.7)	113 (15.3)	** < 0.001**
Calcium channel blocker	81 (23.8)	148 (20.0)	0.173
Direct-acting oral anticoagulant	52 (15.3)	44 (5.9)	** < 0.001**
Insulin	30 (8.8)	36 (4.9)	**0.014**
Loop diuretic	44 (12.9)	34 (4.6)	** < 0.001**
Metformin	71 (20.9)	90 (12.2)	** < 0.001**
Mineralocorticoid receptor antagonist	23 (6.8)	10 (1.4)	** < 0.001**
Sodium-glucose co-transporter 2 inhibitor	10 (2.9)	9 (1.2)	0.077
Statin	185 (54.4)	239 (32.3)	** < 0.001**
**Number of cardiovascular risk factors**			
Less than three	54 (15.9)	246 (33.2)	** < 0.001**
Three to five	211 (62.1)	419 (56.6)	0.097
Six or more	73 (21.5)	66 (8.9)	** < 0.001**

Number of cardiovascular risk factors is based on male sex, age over 65 years, body mass index 25 kg/m^2^ or over, diagnosis of hypertension, dyslipidaemia, diabetes mellitus, and smoking status of patients receiving immune checkpoint inhibitors examining predictors of cardiovascular admissions. Bolded *p*-values indicate statistical significance

*ICI* immune checkpoint inhibitor, *SD* standard deviation, *IQR* interquartile range, *ACEi/ARB* angiotensin converting enzyme inhibitor/angiotensin receptor blocker

Pembrolizumab was the only first-line ICI therapy associated with CV hospitalisation (*p* = 0.045). History of any prior CVD (except VTE) was associated with subsequent CV hospitalisations for patients treated with ICIs (*p* < 0.001, except prior hypertension [*p* = 0.004] and CVaD [*p* = 0.045]). The presence of DM, chronic kidney disease (CKD), and chronic obstructive pulmonary disease (COPD) were also associated with CV hospital admissions (all *p* < 0.001 except CKD [*p* = 0.014]). Consistent with that, prior use of cardiovascular and/or diabetic medications such as angiotensin-converting enzyme inhibitors (ACEi) or angiotensin receptor blockers (ARBs), beta-blockers (BBs), statins, antiplatelets, metformin (all *p* < 0.001), and insulin (*p* = 0.014) was also associated with CV admissions. This was not the case for calcium channel blockers (CCBs) or sodium-glucose co-transporter 2 inhibitors (SGLT2i). The number of CV risk factors and their association with CV hospitalisation are displayed in Table 2. The presence of six or more CV risk factors (*p* < 0.001) were also significantly associated with CV hospitalisation following treatment with ICIs.

Upon multivariable analyses, baseline history of smoking (OR 2.3 [95% CI 1.6–3.3], *p* < 0.001), COPD (OR 1.5 [95% CI 1.0–2.1], *p* = 0.035), arrhythmia (OR 2.2 [95% CI 1.5–3.4], *p* < 0.001), HF/cardiomyopathy (OR 3.6 [95% CI 1.8–7.4], *p* < 0.001), IHD (OR 4.4 [95% CI 3.1–6.4], *p* < 0.001), and prior use of BB (OR 1.5 [95% CI 1.0–2.3], *p* = 0.047) were independently associated with an increased risk of CV hospitalisations. Multivariable analysis for CV hospitalisations results is presented in Table 3.

**Table 3:** Multivariate analysis of variables independently associated with cardiovascular hospitalisations

Variable	Odds ratio	95% CI		*β*-coefficient	*p*-value
		Lower	Upper		
Active or ex-smoker	2.309	1.636	3.259	0.837	** < 0.001**
Age at first ICI Dose	1.024	1.009	1.039	0.024	**0.002**
BMI (kg/m^2^)	1.023	0.999	1.047	0.023	0.059
COPD	1.484	1.027	2.142	0.394	**0.035**
Melanoma cancer diagnosis	1.413	0.968	2.061	0.346	0.073
Other cancer diagnosis	1.452	0.984	2.141	0.373	0.060
Prior arrhythmia	2.221	1.451	3.400	0.798	** < 0.001**
Prior beta-blocker	1.538	1.006	2.349	0.430	**0.047**
Prior diabetes (type I or II)	1.396	0.985	1.978	0.333	0.061
Prior heart failure and/or cardiomyopathy	3.626	1.776	7.402	1.288	** < 0.001**
Prior ischaemic heart disease	4.430	3.060	6.414	1.488	** < 0.001**

Adjusted for age at first immune checkpoint inhibitor (ICI) dose, gender, body mass index (BMI), primary cancer diagnosis (melanoma, cancer of the lung, and other), nivolumab monotherapy, pembrolizumab monotherapy, ipilimumab + nivolumab (including placebo-controlled trial participants), dabrafenib + trametinib combination therapy, prior arrhythmia, prior cerebrovascular disease, prior dyslipidaemia, prior heart failure and/or cardiomyopathy, prior hypertension, prior ischaemic heart disease, prior chronic kidney disease, prior chronic obstructive pulmonary disease, smoking history, diabetes (type I or II), prior angiotensin converting enzyme inhibitor/angiotensin receptor blocker, prior antiplatelet, prior beta-blocker, and prior statin. Bolded *p*-values indicate statistical significance

### Time to first cardiovascular admission

In a cause-specific Cox model for time to first CV admission (*n* = 928; excluding CV admissions with missing admission timing), baseline HF/cardiomyopathy (HR 3.25 [95% CI 1.86–5.67]; *p* < 0.001), arrhythmia (HR 1.75 [95% CI 1.16–2.66]; *p* = 0.008), IHD (HR 1.57 [95% CI 1.04–2.37]; *p* = 0.030), and prior VTE (HR 1.71 [95% CI 1.08–2.70]; *p* = 0.021) were independently associated with earlier CV admission.

### All-cause mortality analysis

A total of 763 patients (70.6%) had died by the conclusion of patient follow-up (31st December 2022). Table 4 illustrates the baseline demographics, comorbidities, cancer diagnosis and staging, and medications associated with mortality in patients treated with ICIs. Patients who died during follow-up were older (mean age 67.8 vs 64.8 years, respectively; *p* < 0.001), with lower BMI (25.6 kg/m^2^ vs 27.8 kg/m^2^, respectively; *p* < 0.001), had a history of VTE (*p* < 0.001), COPD (*p* = 0.042), and statin use (*p* = 0.028). The number of pre-existing CV risk factors was not associated with mortality in our cohort. The days to first CV hospital admission were also significantly different, with patients who were alive at the end of the follow-up period remaining out of hospital for CVD far longer than those who died (644 days [IQR 185–1146] vs 156 days [IQR 55–453] respectively; *p* < 0.001).

**Table 4:** Univariate analysis of patient demographics stratified according to mortality

Characteristic	Alive *n* = 317 (%)	Deceased *n* = 763 (%)	*p*-value
**Patient demographics**			
Age (years) first ICI dose, mean (± SD)	64.8 (± 13.0)	67.8 (± 11.0)	**< 0.001**
Body mass index (kg/m^2^), median (IQR)	27.8 (23.8–30.9)	25.6 (21.9–29.3)	**< 0.001**
Gender (male)	195 (61.5)	490 (64.2)	0.406
Smoking status (active or ex-smoker)	179 (56.5)	490 (64.2)	**0.019**
**Comorbidities**			
Anxiety/depression	66 (28.3)	167 (71.7)	0.745
Arrhythmia	33 (23.6)	107 (76.4)	0.112
Asthma	32 (34.4)	61 (65.6)	0.284
Chronic kidney disease	17 (26.2)	48 (73.8)	0.674
Chronic obstructive pulmonary disease	55 (23.9)	175 (76.1)	**0.042**
Diabetes mellitus (type I or type II)	63 (27.9)	163 (72.1)	0.623
Dyslipidaemia	109 (26.4)	304 (73.6)	0.099
Heart failure and/or cardiomyopathy	11 (20.0)	44 (80.0)	0.130
Hypertension	163 (28.5)	408 (71.5)	0.547
Previous peripheral vascular disease	7 (2.2)	34 (4.5)	0.083
Previous venous thromboembolism	17 (15.0)	96 (85.0)	**< 0.001**
Prior cerebrovascular disease	25 (27.2)	67 (72.8)	0.720
Prior ischaemic heart disease	50 (26.0)	142 (74.0)	0.295
**Cancer type**			
Lung cancer and mesothelioma	62 (19.0)	265 (81.0)	**< 0.001**
Melanoma (includes in situ)	181 (43.3)	237 (56.7)	**< 0.001**
Other cancer types	58 (21.6)	211 (78.4)	**< 0.001**
Unknown/not reported	16 (24.2)	50 (75.8)	0.404
**Cancer staging**			
Stage I	1 (0.3)	14 (1.8)	0.082
Stage II	2 (0.6)	7 (0.9)	1.000
Stage III	60 (18.9)	30 (3.9)	**< 0.001**
Stage IV	204 (64.4)	540 (70.8)	**0.043**
Unknown/not defined	6 (1.9)	21 (2.8)	0.523
**First-line ICI treatment**			
Ipilimumab + nivolumab (includes placebo-controlled clinical trials)	42 (13.2)	42 (5.5)	**< 0.001**
Nivolumab	137 (28.8)	338 (71.2)	0.788
Pembrolizumab	141 (32.9)	287 (67.1)	**0.040**
**Other systemic anticancer treatments**			
Carboplatin	52 (16.4)	298 (39.1)	**< 0.001**
dabrafenib + trametinib	22 (6.9)	54 (7.1)	1.000
Gemcitabine	35 (11.0)	232 (30.4)	**< 0.001**
Paclitaxel	17 (5.4)	60 (7.9)	0.155
**Medications**			
ACEi/ARB	143 (29.2)	346 (70.8)	0.947
Antiarrhythmic	10 (18.2)	45 (81.8)	0.068
Antidepressant	55 (24.1)	173 (75.9)	0.059
Antiplatelet	74 (27.1)	199 (72.9)	0.357
Antipsychotic	8 (25.0)	24 (75.0)	0.696
Beta-blocker	61 (24.6)	187 (75.4)	0.068
Calcium channel blocker	66 (28.8)	163 (71.2)	0.870
Direct-acting oral anticoagulant	24 (25.0)	72 (75.0)	0.350
Insulin	20 (30.3)	46 (69.7)	0.889
Loop diuretic	16 (20.5)	62 (79.5)	0.093
Metformin	42 (26.1)	119 (73.9)	0.349
Mineralocorticoid receptor antagonist	6 (18.2)	27 (81.8)	0.177
Sodium-glucose co-transporter 2 inhibitor	5 (26.3)	14 (73.7)	1.000
Statin	108 (25.5)	316 (74.5)	**0.028**
**Number of cardiovascular risk factors**			
Less than three	99 (0.33)	201 (67.0)	0.117
Three to five	178 (28.3)	452 (71.7)	0.378
Six or more	37 (26.6)	102 (73.4)	0.486

*ICI* immune checkpoint inhibitor, *SD* standard deviation, *IQR* interquartile range, *ACEi/ARB* angiotensin converting enzyme inhibitor/angiotensin receptor blocker. Number of cardiovascular risk factors is based on male sex, age over 65 years, body mass index 25 kg/m^2^ or over, diagnosis of hypertension, dyslipidaemia, diabetes mellitus, and smoking status of patients receiving immune checkpoint inhibitors examining predictors of cardiovascular admissions. Bolded *p*-values indicate statistical significance

On multivariable analysis, CV hospitalisation was independently associated with an increased risk of mortality (OR 2.1 [95% CI 1.5–3.0], *p* < 0.001). Alternatively, factors independently associated with lower mortality risk included first-line ICI treatment with pembrolizumab (OR 0.5 [95% CI 0.3–0.7], *p* < 0.001) and a primary diagnosis of melanoma (OR 0.4 [95% CI 0.3–0.6], *p* < 0.001). Multivariable analysis for mortality outcomes results is presented in Table 5.

**Table 5:** Multivariate analysis of variables independently associated with mortality

Variable	Odds ratio	95% CI		*β*-coefficient	*p*-value
		Lower	Upper		
Age at first ICI dose	1.018	1.005	1.031	0.018	**0.005**
BMI (kg/m^2^)	0.968	0.946	0.991	−0.032	**0.006**
CV hospital admission	2.127	1.516	2.985	0.755	** < 0.001**
Ipilimumab + nivolumab (including placebo-controlled trial)	0.615	0.350	1.081	−0.486	0.091
Nivolumab monotherapy	0.635	0.399	1.012	−0.454	0.056
Pembrolizumab monotherapy	0.453	0.288	0.710	−0.793	** < 0.001**
Primary diagnosis of melanoma (including melanoma in situ)	0.424	0.308	0.584	−0.858	** < 0.001**
Prior venous thromboembolism	1.694	0.965	2.975	0.527	0.067
Stage III cancer diagnosis	0.180	0.108	0.299	−1.717	** < 0.001**

Adjusted for cardiovascular hospital admission, age at first immune checkpoint inhibitor (ICI) dose, gender, body mass index (BMI), primary cancer diagnosis (melanoma, cancer of the lung, other), cancer staging, nivolumab monotherapy, pembrolizumab monotherapy, ipilimumab + nivolumab (including placebo-controlled trial participants), prior arrhythmia, prior cerebrovascular disease, prior dyslipidaemia, prior heart failure and/or cardiomyopathy, prior hypertension, prior ischaemic heart disease, prior venous thromboembolism, prior chronic kidney disease, prior chronic obstructive pulmonary disease, prior asthma, smoking history, anxiety/depression, diabetes (type I or II), prior angiotensin converting enzyme inhibitor/angiotensin receptor blocker, prior beta-blocker, and prior statin. Bolded *p*-values indicate statistical significance.

### Survival analysis

**Fig. 1:**
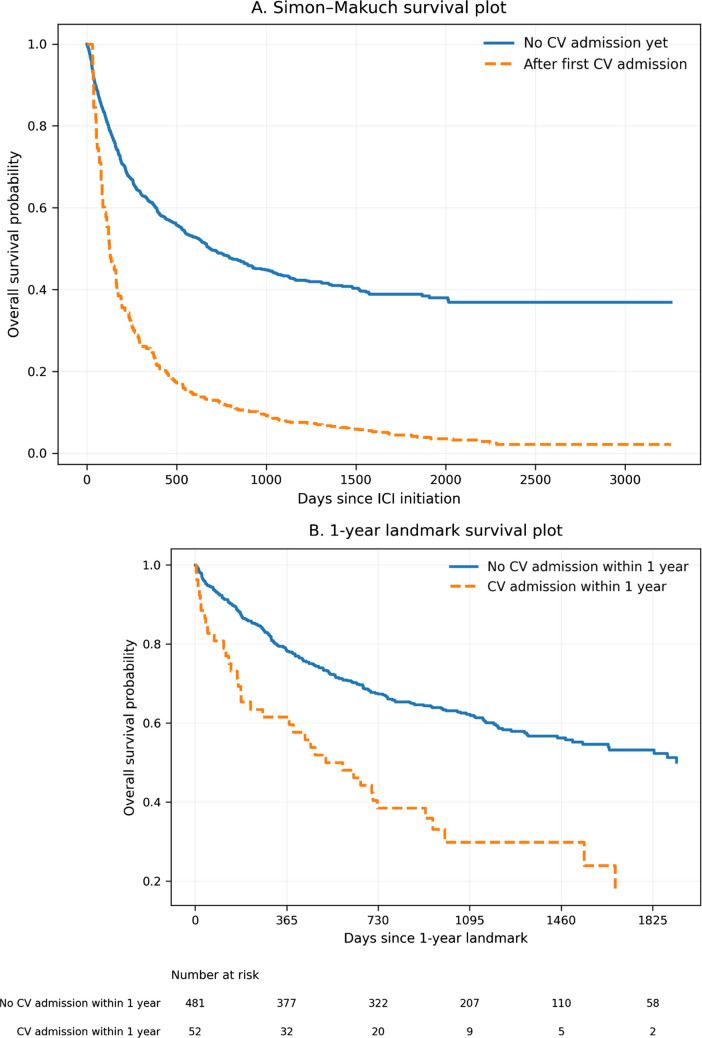
Survival analyses demonstrating the association between cardiovascular hospital admission and subsequent all-cause mortality in patients treated with immune checkpoint inhibitors. **A** Simon–Makuch survival plot showing survival according to the time-varying occurrence of first cardiovascular hospital admission following immune checkpoint inhibitor initiation. **B** One-year landmark Kaplan–Meier survival analysis among patients alive at 365 days, stratified by whether a cardiovascular hospital admission occurred within the first year

In time-dependent Cox models treating CV admission as a time-varying covariate (*n* = 928; excluding CV admissions with missing admission timing), the hazard of death was higher after the first CV admission compared with before admission (and compared with those never admitted). The association was consistent in unadjusted analyses (HR 3.33 [95% CI 2.75–4.02]; *p* < 0.001), age- and sex-adjusted analyses (HR 3.31 [95% CI 2.73–4.01]; *p* < 0.001), and a fully adjusted model (HR 3.29 [95% CI 2.70–4.02]; *p* < 0.001). These findings are illustrated in the Simon–Makuch plot** (**Fig. 1A**).**

As a complementary sensitivity analysis, a 1-year landmark analysis restricted to patients alive at 365 days (*n* = 533) showed that CV admission within the first year was associated with higher subsequent mortality (unadjusted HR 2.37 [95% CI 1.67–3.37]; *p* < 0.001; adjusted HR 2.36 [95% CI 1.61–3.45]; *p* < 0.001). The landmark survival curve is shown in Fig. 1B.

## Discussion

In this large Australian real-world cohort, we identify baseline factors associated with subsequent cardiovascular hospitalisation and all-cause mortality among patients treated with immune checkpoint inhibitors and describe several cardiovascular events beyond myocarditis. The most salient findings are that prior history of arrhythmia increases the risk of CV hospitalisation over twofold, while pre-existing HF and/or cardiomyopathy or IHD increases that risk around fourfold, in patients subsequently treated with ICIs. Our results are consistent with current evidence that reports high rates of CV risk factors in people receiving cancer treatment and provides further insight into the increased frequency of hospital readmissions for atrial fibrillation and IHD observed in this population [20].

The bidirectional relationship between cancer and CVD has been extensively reported within the literature, which includes shared modifiable risk factors such as hypertension and dyslipidaemia, along with inflammation and genetic susceptibility [21–27]. This predisposes people with cancer to an increased risk of CVD development [28]. Subsequent commencement of potentially cardiotoxic anticancer therapy imparts further risk of CV complications in people with cancer [29]. The association between cancer chemotherapy, antecedent CVD and subsequent mortality has previously been described in a large retrospective study of over 17,000 Australians with a history of cancer [30]. Our results extend upon this research by demonstrating a similar association with ICI therapy which has not been previously described.

Historically, the focus of literature exploring ICI-related CV complications and associated risk factors has been on the development and management of myocarditis [9, 11, 31, 32]. Although the development of myocarditis carries poor prognosis and mortality rates range between 25 and 50%, the incidence of ICI-related myocarditis is rare, with reported rates between 0.04% and 1.14% [9]. Emerging evidence also focusses on ICI association with accelerated atherosclerotic plaque development [33, 34]: a recent study demonstrated a 4.7-fold increase in atherosclerotic CV events associated with ICI therapy [11]. Others have observed an approximately 1% incidence of acute vascular complications and arterial thrombotic events in patients treated with ICIs [35, 36]. Recently, a retrospective analysis of 366 patients with cancer receiving ICI therapy reported a 7.1% incidence of atherosclerotic CVD events over a median of 3.4-year follow-up [37].

Our results suggest the risk of hospitalisation secondary to CVD is a much more common issue for cancer patients who receive ICIs. Nearly one-in-three patients in our cohort had at least one CV hospitalisation following ICI treatment, with pre-existing arrhythmia, HF and/or cardiomyopathy, and IHD being the dominant risk factors for CV admissions. These risk factors closely mirrored the reasons for CV hospitalisation post-ICI therapy, with nearly one-in-five CV hospital admissions attributed to IHD, and nearly one-in six CV hospital admissions attributed to atrial fibrillation or atrial flutter. This is compared to our reported myocarditis-related hospital admission prevalence of 1.8%.

CVD hospitalisation is also associated with significant financial burden. During the 2020–21 financial year in Australia, an estimated 9.5% of total allocated health system expenditure was attributed to CVD ($14.3 billion AUD) [38]. Of this, 65% or $9.2 billion AUD was spent on hospital services. The high incidence of CV hospitalisation in cancer patients receiving ICI therapy may potentially impart a more significant burden on healthcare systems. Pharmacist-led interventions can significantly reduce the risk factors of CV events, reducing healthcare system costs and patient burden [39]. We have previously described the role of the pharmacist as part of the cardio-oncology multidisciplinary team (MDT) and postulated their role in CV risk factor management across the entire cancer care continuum [40]. Pharmacists are ideally positioned to provide transitional care services including tailored patient education, medication reconciliation and review, and CV risk factor screening initiatives [41]. As such, greater implementation of pharmacists within the cardio-oncology MDT may reduce CV-associated morbidity in people with cancer receiving ICIs.

Fewer factors, however, were associated with all-cause mortality in our study population. Prior VTE, COPD, and statin use were all significant predictors of all-cause mortality on univariate analysis, although these variables did not reach statistical significance on multivariate analysis. A primary diagnosis of melanoma and first-line ICI treatment with pembrolizumab monotherapy were significantly associated with lower risk of all-cause mortality on multivariate analysis. These results may likely reflect the more favourable survival estimates in ICI-treated patients diagnosed with metastatic melanoma and treated with pembrolizumab compared to metastatic lung cancer [42–44].

It is well-documented that cancer survivors who subsequently develop CVD exhibit higher mortality rates [45–47]. In analyses accounting for the time-dependent nature of CV hospitalisation, we observed a marked increase in the hazard of death after the first CV admission in patients treated with ICIs (fully adjusted time-dependent HR 3.29). The Simon–Makuch plot (Fig. 1A) visually complements this finding by demonstrating lower survival probability following first CV admission when analysed as a time-varying exposure. In a complementary 1-year landmark analysis restricted to patients surviving beyond the first year, CV admission within the first year remained associated with higher subsequent mortality (adjusted HR 2.36; Fig. 1B). This evidence reinforces the need for appropriate monitoring and management of pre-existing CVD prior to commencing ICI therapy with ongoing surveillance during treatment and throughout the survivorship phase of the disease.

### Strengths and limitations

We report the risk factors associated with CV hospitalisations and all-cause mortality in people treated with ICIs for their cancer. The key strength of this study is the large sample size, with over 1,000 patients included in the study. It is the largest cohort of ICI-treated patients described in Australia and among the largest in the world examining predictors and impact of CV hospitalisations, where intricate clinical data are available. Additionally, our cohort represents a key subset of the Australian population living outside major capital cities.

The primary limitation, associated with any retrospective study, is the reliance on accurate reporting within electronic medical records. The authors acknowledge the possibility of missed or incorrectly reported data impacting our results, including cause of death, CV hospital readmissions and corresponding diagnoses, timing of CV hospitalisations, cancer therapy, including ICI access, and cancer-specific characteristics including brain involvement and disease burden. However, retrospective analysis does provide key insight into possible association, in this case, between CV risk factors, CV hospitalisations, and mortality. The larger sample size within this study helps overcome some of these barriers. Another challenge posed by retrospective analysis is to extract the finer, more granular clinical outcomes or temporal relationships that may only be achieved through prospective observational research. Results from our study therefore help inform future prospective studies to better answer these questions.

The ability to ascertain direct causal relationships between ICI therapy and CV hospitalisations or mortality is challenging without the inclusion of an ICI-free matched cancer cohort. Future retrospective studies would benefit from the inclusion of this comparator cohort to draw more meaningful conclusions between ICI therapy and subsequent CV hospitalisations and mortality.

For this study, we consolidated CV hospitalisations into a single, composite outcome to ensure sufficient statistical power for analysis. However, the authors acknowledge the possible limitations surrounding this methodology, including the variance in pathophysiology, especially within a cancer population, and clinical severity. This may limit interpretability of results without provision of more elaborate, cause-specific primary outcomes. In addition, CV hospitalisation occurs after cohort entry and may function as an intermediate event in relation to mortality. If treated as a fixed baseline covariate in survival analyses, this could introduce survival-time (immortal-time) bias. We addressed this limitation by performing time-dependent Cox models with CV admission as a time-varying covariate and a 1-year landmark sensitivity analysis. However, admission timing was unavailable for a subset of patients with CV admissions, necessitating exclusion of those individuals from analyses requiring time to admission and raising the possibility of selection bias. Future studies with Fine-Gray cumulative incidence methods, complete event timing and competing-risk sub-distribution approaches would provide complementary estimates of cumulative incidence, providing further refining temporal and causal inference.

## Conclusion

In over 1000 patients treated with immune checkpoint inhibitors within an Australian regional health district, baseline cardiovascular disease and risk factors were strongly associated with subsequent cardiovascular hospitalisation and all-cause mortality. Prior IHD, HF and/or cardiomyopathy, and arrhythmia increased the risk of CV hospitalisation up to over fourfold during ICI treatment. In time-dependent and landmark analyses accounting for admission timing, CV hospitalisation was associated with substantially higher subsequent mortality (time-dependent adjusted HR 3.29; 1-year landmark adjusted HR 2.36).

Future research should focus on providing evidence to inform guideline-based recommendations for cancer patients throughout their treatment journey. Emphasis should be placed on best possible management of pre-existing CV risk factors and CVD before, during and after cancer treatment, with the inclusion of cardio-oncology survivorship services. This may, in turn, mitigate the burden of CV hospitalisation and all-cause mortality for people with cancer.

## Supplementary Information

Below is the link to the electronic supplementary material.

ESM 1 (DOCX 36.4 KB)

## Data Availability

All data relevant to the study was included either in the manuscript or as supplementary material.

## Abbreviations

ACEi: Angiotensin-converting enzyme inhibitor
ARB: Angiotensin receptor blocker
AUD: Australian dollar
BB: Beta-blocker
BMI: Body mass index
CAP: *Clinical Applications Portal*
CCB: Calcium channel blocker
CI: Confidence interval
CKD: Chronic kidney disease
COPD: Chronic obstructive pulmonary disease
CTLA-4: Cytotoxic T-lymphocyte-associated antigen 4
CV: Cardiovascular
CVaD: Cerebrovascular disease
CVD: Cardiovascular disease
DM: Diabetes mellitus
HF: Heart failure
HLP: Hyperlipoproteinaemia
HNELHD: *Hunter New England Local Health District*
HR: Hazard ratio
ICD-10: *International Statistical Classification of Diseases and Related Health Problems,* *10th* *Revision*
ICI: Immune checkpoint inhibitor
IHD: Ischaemic heart disease
irAE: Immune-related adverse effect
IQR: Interquartile range
NSCLC: Non-small-cell lung cancer
OR: Odds ratio
PD-1: Programmed death 1
PD-L1: Programmed cell death-ligand
PVD: Peripheral vascular disease
SGLT2i: Sodium-glucose co-transporter 2 inhibitor
SD: Standard deviation
VTE: Venous thromboembolism
