# Supplementary material for: Risk factors associated with cardiovascular hospital admissions and all-cause mortality in cancer patients treated with immune checkpoint inhibitors
Source: Support Care Cancer. 2026 Jul 17;34(8):770. doi: 10.1007/s00520-026-10983-6 (PMC13375742; doi:10.1007/s00520-026-10983-6)
Supplement: Supplementary file 1 — (DOCX 36.4 KB) [file 520_2026_10983_MOESM1_ESM.docx]

**Supplementary Tables**

**Supplementary Table 1:** Follow-up information, number of different immune checkpoint inhibitors and other cancer treatments used in patients treated with immune checkpoint inhibitors.

| **Characteristic** | **General Cohort**  ***n* = 1,080 (%)** |
| --- | --- |
| **Follow-Up** | |
| Follow-Up Time (Years) After ICI Exposure, median (IQR) | 1.4 (0.4–3.7) |
| Deceased During Follow-Up | 763 (70.6) |
| Age (Years) Deceased, mean (± SD) | 68.9 (± 11.1) |
| Days to Death Post ICI, median (IQR) | 441.5 (93–592) |
| **Sequential ICI Treatment** | |
| Single ICI | 912 (84.4) |
| Two ICIs | 141 (13.1) |
| Three or more ICIs | 27 (2.5) |

ICI, immune checkpoint inhibitor; SD, standard deviation; IQR, interquartile range.

**Supplementary Table 2:** Follow-up information, number of different immune checkpoint inhibitors and other cancer treatments used in patients treated with immune checkpoint inhibitors examining predictors of cardiovascualr hospital admission.

| **Characteristic** | **CV Admission**  ***n* = 340 (%)** | **No CV Admission**  ***n* = 740 (%)** | ***p*-value** |
| --- | --- | --- | --- |
| **Follow-Up** | | | |
| Follow-Up Time (Years) After ICI Exposure, median (IQR) | 1.2 (0.5–2.6) | 1.3 (0.5–2.9) | 0.150 |
| Deceased During Follow-Up | 273 (80.3) | 490 (66.2) | **< 0.001** |
| Age (Years) Deceased, mean (± SD) | 70.38 (± 10.2) | 70.6 (± 9.2) | **< 0.001** |
| Days to Death Post ICI, median (IQR) | 440 (176–938.5) | 483.5 (185.25–1,072.5) | **< 0.001** |
| **Sequential ICI Treatment** | | | |
| Single ICI | 284 (83.5) | 628 (84.9) | 0.588 |
| Two ICIs | 4 (6.9) | 97 (13.1) | 1.000 |
| Three or more ICIs | 12 (3.5) | 15 (2.0) | 0.147 |

CV, cardiovascular; ICI, immune checkpoint inhibitor; SD, standard deviation; IQR, interquartile range.

**Supplementary Table 3:** Follow-up information, number of different immune checkpoint inhibitors and other cancer treatments used in people treated with immune checkpoint inhibitors examining predictors of mortality.

| **Characteristic** | **Alive**  ***n* = 317 (%)** | **Deceased**  ***n* = 763 (%)** | ***p*-value** |
| --- | --- | --- | --- |
| **Follow-Up** | | | |
| Follow-Up Time (Years) After ICI Exposure, median (IQR) | 4.5 (3.7–5.6) | 0.6 (0.3–1.7) | **< 0.001** |
| **Sequential ICI Treatment** | | | |
| Single ICI | 253 (27.7) | 659 (72.3) | **0.010** |
| Two ICIs | 54 (38.3) | 87 (61.7) | **0.017** |
| Three or more ICIs | 10 (37.0) | 17 (63.0) | 0.394 |

ICI, immune checkpoint inhibitor; SD, standard deviation; IQR, interquartile range.

**Supplementary Table 4:** Primary diagnosis of first cardiovascular disease hospital admission amongst cancer patients following their first dose of immune checkpoint inhibitors.

| **Primary CV Diagnosis** | **CV Admission**  ***n* = 340 (%)** |
| --- | --- |
| Acute Coronary Syndromes | 7 (2.1) |
| Acute Myocardial Infarction | 25 (7.4) |
| Atrial Fibrillation and Atrial Flutter | 53 (15.6) |
| Cardiovascular (Other/Not Specified) | 43 (12.6) |
| Cerebrovascular Disease | 34 (10.0) |
| Heart Failure | 39 (11.5) |
| Ischaemic Heart Disease (Not Specified) | 66 (19.4) |
| Myocarditis | 6 (1.8) |
| Pericarditis | 2 (0.6) |
| Venous Thromboembolism | 65 (19.1) |

Other cardiovascular disease admission includes valvular heart disease, pulmonary hypertension, and other conduction disorders. CV, cardiovascular.

**Supplementary Figures**

**Supplementary Figure 1:** Consort diagram of patients treated with immune checkpoint inhibitors included in the final analysis. ICI, immune checkpoint inhibitor; HNELHD, *Hunter New England Local Health District*.

Total patients identified as receiving ICIs between 1^st^ January 2010 and 1^st^ January 2020 in the HNELHD

*n* = 1,129

*N*=1,129

Patients treated with ICIs followed up across study period

*n* = 1,085

**Patients included in the final analysis**

*n* = 1,080

Patients not prescribed ICIs

*n* = 44

Incomplete treatment data

*n* = 5
